# Autosomal Recessive Transmission of *MYBPC3* Mutation Results in Malignant Phenotype of Hypertrophic Cardiomyopathy

**DOI:** 10.1371/journal.pone.0067087

**Published:** 2013-06-28

**Authors:** Yilu Wang, Zhimin Wang, Qi Yang, Yubao Zou, Hongju Zhang, Chaowu Yan, Xinxing Feng, Yi Chen, Yin Zhang, Jizheng Wang, Xianliang Zhou, Ferhaan Ahmad, Rutai Hui, Lei Song

**Affiliations:** 1 Department of Cardiology, State Key Laboratory of Cardiovascular Disease, Fuwai Hospital, National Center for Cardiovascular Disease, Chinese Academy of Medical Sciences and Peking Union Medical College, Beijing, China; 2 Department of Ultrasound, Fuwai Hospital, Chinese Academy of Medical Sciences and Peking Union Medical College, Beijing, China; 3 Radiology Department, Xuanwu Hospital, Capital Medical University, Beijing, China; 4 Department of Radiology, Fuwai Hospital, Chinese Academy of Medical Sciences and Peking Union Medical College, Beijing, China; 5 Endocrinology and Cardiovascular Center, Fuwai Hospital, Chinese Academy of Medical Sciences and Peking Union Medical College, Beijing, China; 6 Surgical ICU, Fuwai Hospital, Chinese Academy of Medical Sciences and Peking Union Medical College, Beijing, China; 7 Sino-German Laboratory for Molecular Medicine, State Key Laboratory of Cardiovascular Disease, Fuwai Hospital, National Center for Cardiovascular Disease, Chinese Academy of Medical Sciences and Peking Union Medical College, Beijing, China; 8 Hypertension Center, State Key Laboratory of Cardiovascular Disease, Fuwai Hospital, National Center for Cardiovascular Disease, Chinese Academy of Medical Sciences and Peking Union Medical College, Beijing, China; 9 Cardiovascular Genetics Center and Hypertrophic Cardiomyopathy Center, UPMC Heart and Vascular Institute, University of Pittsburgh, Pittsburgh, Pennsylvania, United States of America; Innsbruck Medical University, Austria

## Abstract

**Background:**

Hypertrophic cardiomyopathy (HCM) due to mutations in genes encoding sarcomere proteins is most commonly inherited as an autosomal dominant trait. Since nearly 50% of HCM cases occur in the absence of a family history, a recessive inheritance pattern may be involved.

**Methods:**

A pedigree was identified with suspected autosomal recessive transmission of HCM. Twenty-six HCM-related genes were comprehensively screened for mutations in the proband with targeted second generation sequencing, and the identified mutation was confirmed with bi-directional Sanger sequencing in all family members and 376 healthy controls.

**Results:**

A novel missense mutation (c.1469G>T, p.Gly490Val) in exon 17 of *MYBPC3* was identified. Two siblings with HCM were homozygous for this mutation, whereas other family members were either heterozygous or wild type. Clinical evaluation showed that both homozygotes manifested a typical HCM presentation, but none of others, including 5 adult heterozygous mutation carriers up to 71 years of age, had any clinical evidence of HCM.

**Conclusions:**

Our data identified a *MYBPC3* mutation in HCM, which appeared autosomal recessively inherited in this family. The absence of a family history of clinical HCM may be due to not only a de novo mutation, but also recessive mutations that failed to produce a clinical phenotype in heterozygous family members. Therefore, consideration of recessive mutations leading to HCM is essential for risk stratification and genetic counseling.

## Introduction

Hypertrophic cardiomyopathy (HCM) is the most common inherited heart disease and one of the common cause of sudden cardiac death (SCD) [1]. Most cases of HCM are caused by mutations in the genes encoding sarcomere proteins in a Mendelian autosomal dominant pattern [1]–[3]. Genetic testing of these genes in HCM patients has been recommended in the latest guidelines, because of its significant value in diagnosis and early identification of individuals who are at risk, especially among family members [4], [5]. However, nearly 50% HCM patients had no apparent clinical family history of HCM. Although de novo mutations [6], [7] varied clinical penetrance, and the presence of second mutation can attribute to parts of these cases [8]–[13], recessive inheritance may be also involved.

## Methods

### Subjects and Clinical Evaluation

The proband and his family were recruited at Beijing Fuwai Hospital, Chinese Academy of Medical Sciences. Physical examinations, resting and exercise stress M-mode, 2-D, and Doppler echocardiograms, 12-lead ECGs, 24-hour Holter ECGs, and cardiac magnetic resonance imaging (CMR) with late enhancement of gadolinium (LGE) were performed for thorough phenotype characterization of each family member. Three hundred and seventy six individuals with normal ECGs and echocardiograms were also included as healthy controls.

This study was performed in accordance with the principle of the Declaration of Helsinki and approved by the Ethics Committees of Fuwai Hospital. Written informed consents were provided by this family and the healthy controls.

### Genetic Analysis

Genomic DNA was extracted from peripheral blood leukocytes [14]. In the proband, the entire coding sequence and the flanking regions of 26 HCM-related genes, including *MYH7, MYBPC3, TNNT2, TNNI3, MYL2, MYL3, TPM1, ACTC1, MYH6, TNNC1, TTN, ACTN2, TCAP, VCL, ANKRD1, CAV3, CSPR3, LDB3, MYOZ2, NEXN, JPH2, PLN, CASQ2, CALR3, PRKAG2* and *LAMP2,* were enriched by using a custom designed library (Agilent Technologies, Santa Clara, CA, USA), and subsequently sequenced on Genome Analyzer IIx (Illumina Inc, CA, USA). The variant was considered as disease-causing mutation if it was absent in the genetic database of 307 Chinese healthy controls, in which the 26 HCM-related genes were completely screened in the same manner as did in the proband. The identified mutation in the proband was then assessed in all family members and the other 376 healthy controls with bi-directional Sanger sequencing after PCR amplification of corresponding exon. Previous reports of the mutations in public polymorphism databases were determined by searching dbSNP and 1000 Genomes at http://www.ncbi.nlm.nih.gov/projects/SNP and http://www.1000genomes.org, respectively. The pathogenicity of the mutation was predicted with PolyPhen 2 and SIFT [15], [16]. Protein sequence homology of mutation-affected regions among species was determined with Clustal W2 [17].

## Results

### Proband

The proband (III-2, 21 years old) was referred for cardiac evaluation after the SCD of his older brother (III-1) at 23 years of age, who had been diagnosed HCM in another hospital but had not been offered an implantable cardioverter defibrillator (ICD) because of the absence of clinical symptoms or family history (medical record was not available). The proband complained about mild chest pain after intense exertion over the past two years. His ECG showed diffuse repolarization changes with inverted T waves, transthoracic echocardiogram showed mid to distal interventricular septal hypertrophy and CMR showed hypertrophy of the mid to distal interventricular septum and the inferior ventricular wall (Table 1).

**Table 1:** Genotypes and clinical characteristics of all family members.

						Echocardiogram								CMR			
Subject Number	Age (yr)	Mutation Type	Symptoms	Medical History	Blood Pressure (mmHg)	LVEDD (mm)	IVS (mm)	LVPW (mm)	LVEF (%)	LA Diameter (mm)	SAM	LVOT obstruction (mmHg)	ECG Findings (including Holter)	IVS (mm)	LVPW (mm)	Apex (mm)	LGE
**I-1**	68	**Wild Type**	No	**HT 20 years**	170/100	43	17	15	64	36	No	No	Normal	N.A.			
**I-2**	71	Heterozygous	No	No	140/80	46	10	10	61	35	No	No	Normal	9	9	5	No
**I-3**	62	Heterozygous	Chestpain	CAD 6 years	140/80	47	10	10	65	30	No	No	Normal	8	8	6	No
**I-4**	61	Wild Type	No	No	130/80	39	9	10	66	30	No	No	Normal	N.A.			
**II-1**	44	Heterozygous	No	No	120/75	46	9	8	62	32	No	No	Normal	8		5	No
**II-2**	42	Heterozygous	No	No	130/80	47	9	8	57	32	No	No	Normal	7	6	5	No
**II-3**	37	Heterozygous	No	No	140/100	52	11	10	67	34	No	No	Normal	8	7	5	No
**II-4**	29	Wild Type	No	No	120/86	52	10	10	64	36	No	No	Normal	N.A.			
**III-2**	21	Homozygous	Chest pain	No	100/60	55	18	9	66	39	No	No	Diffuse repolarization changes with inverted T waves; premature ventricular contractions	17	10	8	No
**III-3**	19	Homozygous	No	No	100/60	46	15	9	71	34	No	No	Diffuse repolarization changes with inverted T waves	13	9	12	No
**III-4** *	8	Heterozygous	No	No	95/60	38	5	6	67	38	No	No	Normal	N.A.			
**III-5**	4	Wild Type	No	No	N.A.	31	6	6	68	21	No	No	Normal	N.A.			

LVEDD, left ventricular end-diastolic diameter; IVS, inter ventricular septum; LVPW, left ventricular posterior wall; LVEF, left ventricular ejection fraction; LA, left atrium; SAM, systolic anterior motion; LVOT, left ventricular outflow tract; CMR, cardiac magnetic resonance imaging; LGE, late enhancement of gadolinium; ECG, electrocardiographic; HT, hypertension; CAD, coronary artery disease; N.A., not applicable.

* LGE was not performed on III-4, because we considered that it was not necessary to perform an invasive examination at this young age.

### Family History

**Figure 1:**
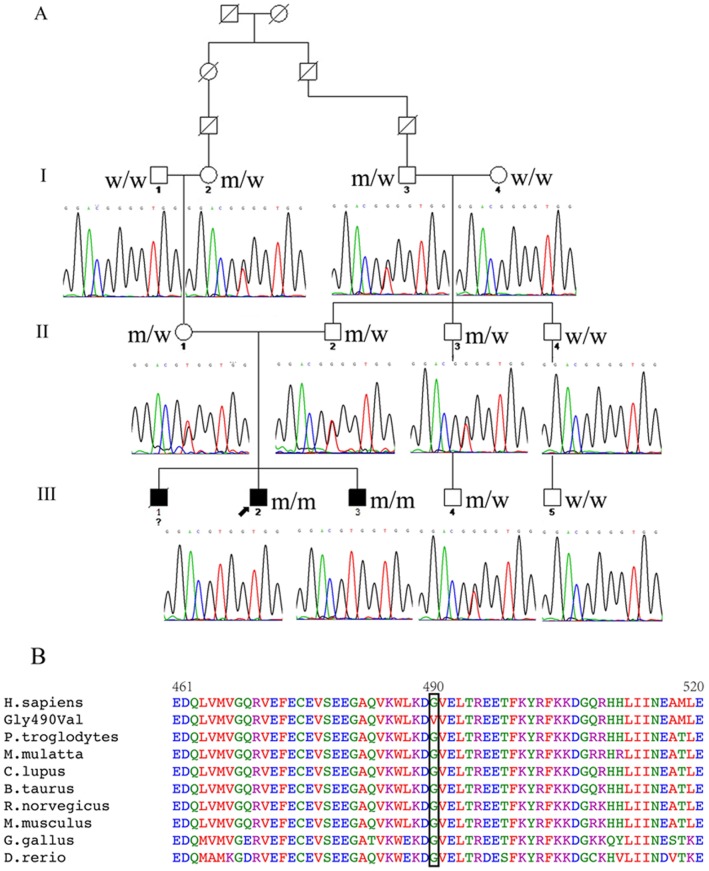
Pedigree of the family with the mutation c.1469G>T (p.Gly490Val) in *MYBPC3* (A). Square, male; circle, female; empty, absent of clinical findings; black, clinically affected; “w”, wild-type allele; ‘m’, mutant allele; ?, no genetic testing performed; black arrow, proband. Protein sequence homology of mutation-affected regions among species (B), determined using Clustal W2. The Gly490Val substitution involves an amino acid that is highly conserved among species.

A detailed family history revealed that the proband’s paternal grandfather (I-3) had a 6-year history of coronary heart disease with chest pain. There was no cardiovascular symptoms or medical history identified in other family members. The proband’s parents (II-1 and 2) were found to have a consanguineous relationship (Figure 1A).

### Genotype

A total of 33 nonsynonymous variants were detected in the proband (Table S1). All the variants were present in the genetic database of 307 controls, except a homozygous mutation c.1469G>T within exon 17 of *MYBPC3*, which resulted in a replacement of glycine at the 490^th^ amino acid by valine (p. Gly490Val). This novel mutation was absent from the other 376 healthy controls, and was not previously reported in the dbSNP and 1000 Genomes public polymorphism databases. Sequence comparisons revealed that the amino acid Gly490 is highly conserved among species (Figure 1B), localized in an immunoglobulin domain on MYBPC3 protein. Both PolyPhen 2 and SIFT predicted that this mutation was pathogenic.

Genetic screening of family members showed that the proband’s younger brother (III-3, 19 years old) was also homozygous and 6 other relatives (I-2 and 3, II-1 to 3, III-4), including the proband’s parents, were heterozygous for Gly490Val mutation. All other family members were normal at the 490^th^ codon of *MYBPC3* (Figure 1A).

### Genotype-Phenotype Correlation

**Figure 2:**
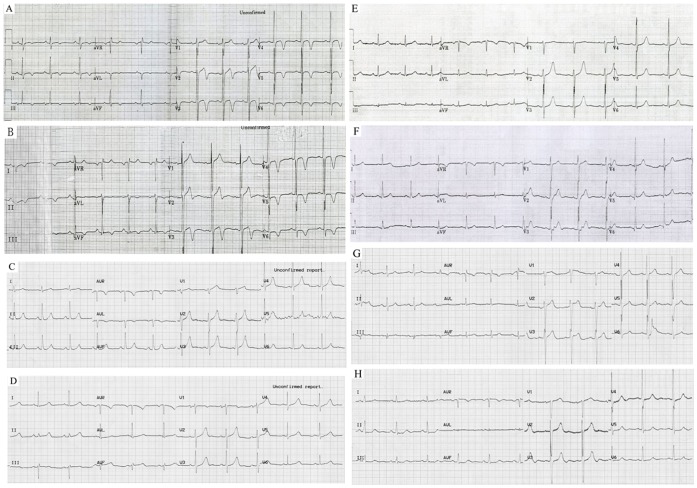
ECGs of proband (III-2) and his younger brother (III-3) (A&B), both of which show diffuse repolarization changes with large negative T waves. ECGs of I-2, I-3 and II-1 to 3 (C to G), five heterozygous mutation carriers in the oldest generation, were normal. ECG of I-1 (H), a wild type family member with 20-year uncontrolled hypertension history, whose echocardiogram showed concentric hypertrophy, was normal.

**Figure 3:**
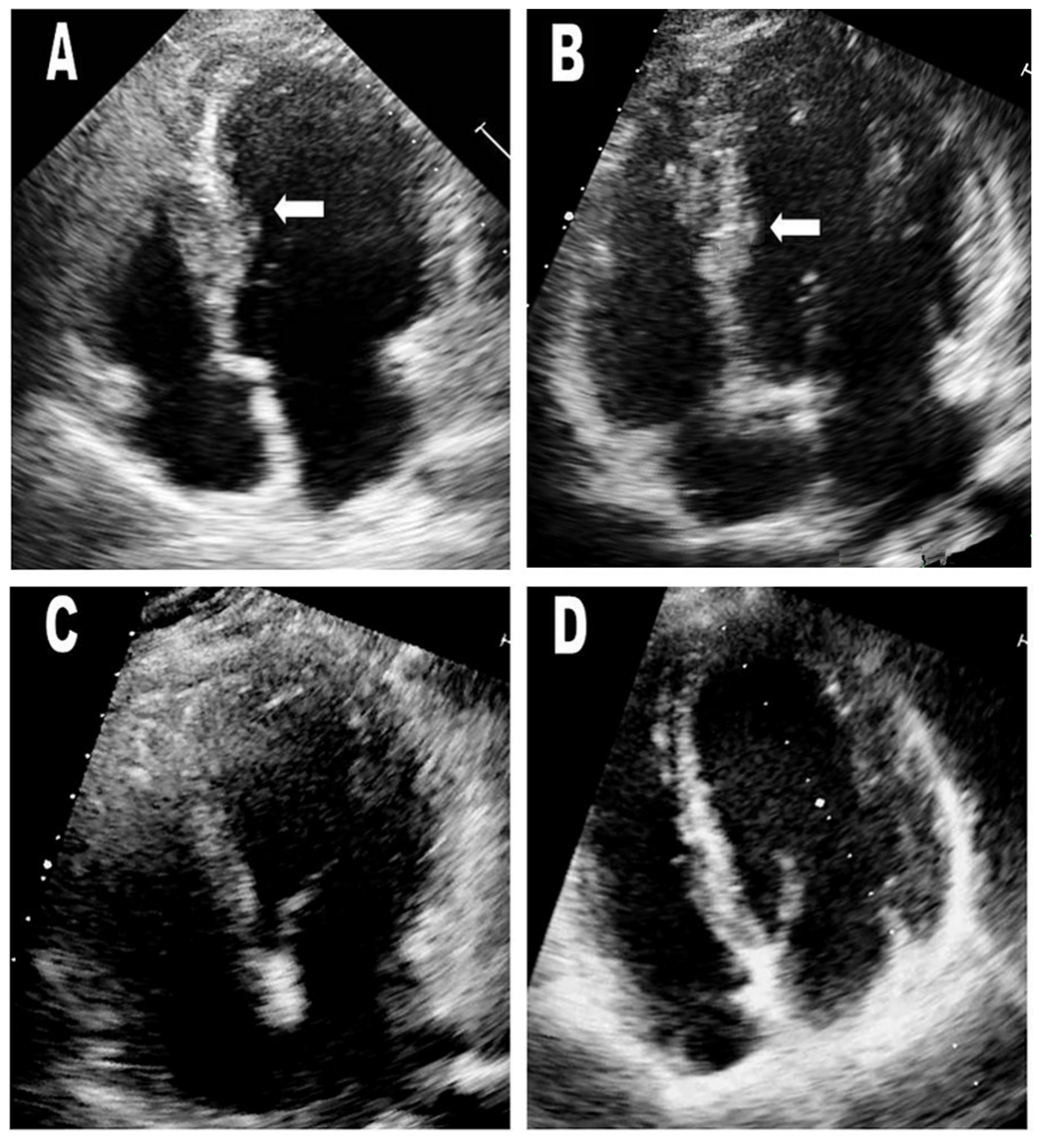
Echocardiograms of the proband (III-2) and his younger brother (III-3) (A&B). White arrows indicate areas of hypertrophy. Maximum wall thicknesses were 18 mm in the proband and 17 mm in his younger brother. Echocardiograms of heterozygous mutation carriers (I-2 and I-3) in the oldest generation were normal (C&D). White arrows indicate the interventricular septum.

**Figure 4:**
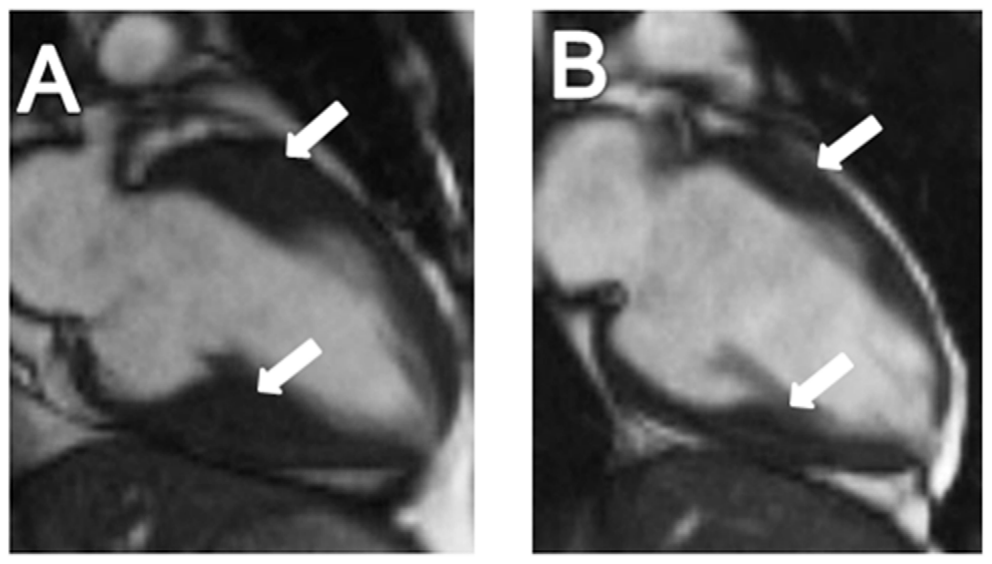
Cardiac magnetic resonance imaging (CMR) of the proband (III-2) (A) and his younger brother (III-3) (B). White arrows indicate areas of hypertrophy in the proband and asymmetrically hypertrophic and stiff ventricular wall in the younger brother (III-3).

In order to confirm whether the Gly490Val mutation causes HCM as an autosomal recessive trait, family members underwent thorough clinical evaluations to detect the presence of HCM (Table 1). Only the two homozygotes exhibited a typical HCM phenotype, including inverted T waves on ECG (Figures 2A and 2B), hypertrophy of the mid to distal interventricular septum on echocardiography (Figures 3A and 3B). CMR showed hypertrophy of the mid to distal interventricular septum and inferior ventricular wall in the proband, and isolated hypertrophic septum and inferior ventricular wall in his younger brother (Figures 4A and 4B). Both of the two homozygotes showed preserved cardiac function (left ventricular ejection fraction, 66% and 71%, respectively), normal atrial and ventricular chamber dimensions, no left ventricular outflow tract (LVOT) obstruction at rest and after exercise, and negative LGE.

All the heterozygous mutation carriers (I-2 and I-3, II-1 to II-3, III-4) showed no typical clinical manifestations of HCM (Table 1). Even the oldest heterozygous mutation carriers (I-2 and I-3), who were 71 and 62 years old, respectively, had no evidence of ECG abnormalities (Figures 2C and 2D) or left ventricular hypertrophy on echocardiogram (Figures 3C and 3D) and CMR. The proband’s parents (II-1 and II-2) and one of his paternal uncles (II-3) did not show any left ventricular hypertrophy nor LVOT obstruction even after exercise provocation, or any arrhythmias on 24-hour Holter. CMR of II-1 to 3 showed neither structural abnormalities nor cardiac fibrosis.

All family members without Gly490Val mutation had normal ECGs and echocardiograms (Table 1), except the maternal grandfather (I-1), who showed concentric hypertrophy on echocardiogram with 17 mm inter ventricular septum and 15 mm left ventricular posterior wall without abnormal T or Q waves or any arrhythmia. However, he had a history of uncontrolled hypertension (170/100 mmHg at enrollment) for greater than 20 years. This type of left ventricular hypertrophy is a typical cardiac remodeling resulted from uncontrolled hypertension.

## Discussion

Taking the advantage of second generation sequencing, we were able simultaneously to screen mutations in 26 known HCM pathogenic genes, and identified a patient carrying a homozygous mutation in *MYBPC3* without an apparent family history of clinical HCM. Autosomal recessive inheritance pattern of HCM due to this *MYBPC3* mutation was supported by the following findings: (1) two clinically affected family members homozygous for the mutation were born to clinically unaffected parents; (2) the parents were consanguineous and heterozygous carriers of *MYBPC3* mutation; (3) all the adult family members who were heterozygous for the mutation did not have a clinically apparent HCM phenotype, even into their 70 s; (4) the family members who harbored homozygous mutations expressed early-onset HCM. Therefore, in some patients with no apparent family history of HCM, an autosomal recessive pattern may be responsible for disease.

MYBPC3 is a crucial component of the sarcomere and an important regulator of muscle function. Among three different MYBPC proteins, *MYBPC3* is expressed exclusively in cardiac myocytes [18], [19] and its HCM-causing mutations were first reported in 1995 [20]. Homozygous mutation in HCM was firstly reported by Ho CY et al in 2000. They described an homozygous Ser179Phe mutation in *TNNT2* gene which caused a severe form of HCM with striking morphological abnormalities and juvenile lethality [21]. From then on, more homozygous mutations were recognized either in case reports or in cohort studies. The inheritance traits were all autosomal dominant because the heterozygotes showed affirmatory but milder clinical evidence of HCM than the homozygotes [21]–[23]. In 2002, the first autosomal recessive transmission of HCM was reported in a family with Glu143Lys mutation in *MYL2* gene. Abnormalities in echocardiogram and ECG were only found in homozygous but not in heterozygous family members [24]. Recently Gray B et al reported a Arg162Trp mutation in *TNNI3* gene could also cause recessive HCM, but lack of clinical and genetic evaluation of old family members [25]. *MYBPC3* is one of the most common disease-causing gene of HCM, accounting for 40–50% of known genetic causes of HCM patients, much higher than the frequency of mutation in *MYL2* (<3%) and TNNI3 (<6%). HCM caused by *MYBPC3* mutations usually manifest lower penetrance, later onset of disease and milder forms of disease progression in comparison to other gene mutations (i.e., *MYH7*) [26], [27]. Patients with multiple mutations (i.e., compound or double heterozygotes) suffer more severe phenotypes and increased risk of SCD [8], [10], [11], [21]. Therefore, we postulated that some *MYBPC3* mutations are functionally so mild that they do not lead to disease unless they are homozygous. In the present study, we screened the *MYBPC3* gene and identified a novel mutation which appeared autosomal recessive inheritance pattern, in a manner, supported the speculation above.

The present mutation (Gly490Val) we identified was a novel one in the domain C3 of *MYBPC3*, with small change of side chain and kept the polarity neutral. Another mutation on the same amino acid residue (Gly490Arg) was reported to cause HCM in heterozygote in western population, substituting the small side chain for a bulky side chain and changing the polarity of amino acid residue into basic [8]. Therefore, the structural change of domain of MYBPC3 protein, which extended into the interfilamental space in the motif binds to myosin S2 [28], due to mutation Gly490Arg much more prominent than that due to mutation Gly490Val. This might be the reason why these two kinds of mutations on the same position presented different inheritance patterns.

In the family described here, our documentation of inheritance of HCM as an autosomal recessive trait had clinical implications. The proband’s older brother had HCM in the absence of any other obvious heart diseases, and died of SCD at young age, suggesting that he was a highly suspicious homozygous mutation carrier. Therefore, the implantation of an implantable cardioverter defibrillator was recommended for the two surviving homozygotes in the family, the proband and his younger brother. Heterozygous family members were felt not to require long-term clinical follow-up.

Our results illustrate the complexity of genetic analysis for HCM. For example, “nonpathogenic” variants in HCM-related genes inherited from parents respectively may lead to HCM in the offspring as recessive mutations. Variants found in clinically unaffected individuals are often considered as benign polymorphisms because almost HCM is most commonly inherited as an autosomal dominant trait. However, this strategy risks the missing of recessive disease-causing mutations. This may partly explain why disease-causing mutations were hard to be found in some typical HCM patients and why more than half of the HCM patients do not have obvious family history.

**In conclusion**, our data identified a *MYBPC3* mutation appeared to be an autosomal recessive transmission in HCM and suggest that the inheritance pattern may be more complex than previously thought. In clinical practice, the absence of a family history of clinical HCM may be due to not only a de novo mutation, but also recessive mutations that failed to produce a clinical phenotype in heterozygous family members. Therefore, consideration of recessive mutations leading to HCM is essential for risk stratification and genetic counseling.

## Supporting Information

Table S1 **The nonsynonymous variants found in the proband.**
(XLS)
